# Editorial: Prodrug design and therapeutic applications

**DOI:** 10.3389/fphar.2023.1280190

**Published:** 2023-09-22

**Authors:** Qicai Xiao, Zhengqiu Li, Yanyan Miao, Jiang Xia, Mingyue Wu

**Affiliations:** ^1^ State Key Laboratory of Targeting Oncology, Guangxi Medical University, Nanning, China; ^2^ School of Pharmaceutical Sciences (Shenzhen), Sun Yat-sen University, Guangzhou, China; ^3^ College of Pharmacy, Jinan University, Guangzhou, China; ^4^ School of Medicine, Shanghai Jiao Tong University, Shanghai, China; ^5^ Department of Chemistry, The Chinese University of Hong Kong, Shatin, Hong Kong SAR, China; ^6^ Department of Chemistry, National University of Singapore, Singapore, Singapore

**Keywords:** prodrug, stimuli-responsive, biological activity, bioavailability, therapeutic application

Prodrugs are chemically derived versions of biologically active molecules, which are inherently indolent but capable of being chemically and/or enzymatically converted to their active counterparts upon triggered by endogenous (e.g., reactive oxygen species (ROS), *p*H, enzymes, etc.) and/or external stimulus (e.g., light, thermal, ultrasound, magnetism, etc.). Prodrug-based drug development strategy has become a well-established tool to improve the physiochemical (PC, e.g., stability, solubility, etc.), pharmacokinetic (PK), pharmacodynamics (PD) and pharmaceutical (PH) properties (called 4P properties) of biologically active agents. It is estimated that over 10% of all the marketed drugs to now are prodrugs. Herein, we launch this Research Topic on a relatively small scale to summarize the latest important advances and some newly emerging technologies in the design of prodrugs and their therapeutic applications.

This Research Topic provides 7 articles, including 2 review articles and 5 original research articles contributed by 62 authors worldwide. The reviews cover Research Topics related to prodrugs in traditional Chinese medicinal herbs and nanotechnology. Obesity as a global health problem has attracted widespread attention, and contributes to the occurrence of various diseases including cardiovascular disease, metabolic syndrome, diabetes and certain cancers. Chinese medicinal herbs-derived molecules such as resveratrol, berberine, artemisinin, curcumin and celastrol are found to be potent therapeutics for obesity. However, these naturally derived compounds have limitations including weak aqueous solubility, low bioavailability, poor stability and strong systematic toxicity. Further derivation and/or modification to the corresponding prodrugs of these herbs-based molecules have the potential to address the above mentioned issues and provide improved biological activities (Law et al.).

5-aminolevulinic acid (ALA) is a clinically approved prodrug predominally used in photodynamic therapy (PDT) and fluorescence-assisted photodynamic diagnosis (PDD). Although ALA itself possesses certain advantages such as better aqueous solubility and enhanced stability compared with the parental drug Protoporphyrin IX (PpIX), it has limitations including weak lipophilicity, poor bioavailability and nonselectivity. The recent emerging nanotechnology opens the avenue for amplifying the effects of ALA-based prodrugs in improved PDT (Lou et al.).

Besides, a diverse of original studies on this Research Topic has given various aspects of different prodrug designs and their therapeutic applications. For instance, Xiao et al. designed and synthesized esterase-responsive prodrugs as potent Bruton’s tyrosine kinase (BTK) inhibitors, which provided improved solubility, stability and kinase activity (Xiao et al.). Similarly, Pinzaru et al. prepared nutraceutical prodrugs using Novozyme^®^ 435-catalyzed esterification of Rutin with unsaturated fatty acids. The resulted Rutin bioconjugates possess improved physiochemical properties, as well as enhanced biosafety and bioavailability (Pinzaru et al.).

It is known that lipid-based technology for enhancement of bioavailability has been widely utilized in a number of U.S. Food and Drug Administration (FDA) approved drugs. However, one of the major limitations of current lipid-based prodrugs is the rapid metabolism of fatty acids in the liver, which greatly shortens the duration of action. Toti et al. developed a strategy by the installation of novel lipid terminal motifs featuring various oxygen contents to an acyclic nucleoside phosphonate tenofovir (TFV)-an approved antiviral agent, which showed enhanced metabolic stability while maintaining the antiviral activity of TFV (Toti et al.). Such a strategy indicates that small modifications to alter the metabolic soft spots of lipid prodrugs, can bring substantial improvements in PK profiles, thereby enabling molecules that are otherwise undevelopable as potential therapeutics for human diseases.

Although the stimuli-responsive feature is one of the major advantages of prodrug-based therapeutics, the uncontrolled bioconversion/release of the parental drug from a prodrug often fails to achieve the desired therapeutic effect. In particular, little is known about the bioconversion enzymes that cleave specific bonds in prodrugs. Hugele et al. investigated the possible enzymes and effectors (e.g., pH, metal ions) on bioconversion of L-type amino acid transporter 1 (LAT1) using amide-based prodrugs in different biological media (Hugele et al.). Aminopeptidase B was identified as a potent enzyme for the bioconversion of amide-based prodrugs containing aromatic promoieties (L-Phe), but not for those prodrugs containing aliphatic amino acids. This study is useful for design of siteselective prodrug delivery system and identification of potent organ-selective enzymes in future. Additionally, virtual screening is a computational technology that analyzes libraries of small molecules to identify potential hit candidates. By combination of machine learning activity prediction and virtual screening, followed by efficient *in vitro* and *in vivo* models of activity evaluation, it can also assist in the discovery and design of innovative prodrugs (Wang et al.).

Overall, the current Research Topic summarizes aspects of “*prodrug design and therapeutic applications*”. It is hoped that this interdisciplinary Research Topic will provide useful information and guidelines for academic and industrial researchers to improve drug efficacy, reduce undesirable side effects and develop innovative therapeutics for the treatment of various diseases.

